# The incremental healthcare cost associated with cancer in Belgium: A registry‐based data analysis

**DOI:** 10.1002/cam4.6659

**Published:** 2024-01-24

**Authors:** Vanessa Gorasso, Stefanie Vandevijvere, Johan Van der Heyden, Ingrid Pelgrims, Henk Hilderink, Wilma Nusselder, Claire Demoury, Masja Schmidt, Stijn Vansteelandt, Delphine De Smedt, Brecht Devleesschauwer

**Affiliations:** ^1^ Department of Epidemiology and Public Health Sciensano Brussels Belgium; ^2^ Department of Public Health and Primary Care Ghent University Ghent Belgium; ^3^ Department of Risk and Health Impact Assessment Sciensano Brussels Belgium; ^4^ Department of Applied Mathematics, Computer Science and Statistics Ghent University Ghent Belgium; ^5^ Centre for Public Health Forecasting National Institute for Public Health and the Environment (RIVM) Utrecht The Netherlands; ^6^ Department of Public Health Erasmus Medical Center Rotterdam The Netherlands; ^7^ Department of Translational Physiology Infectiology and Public Health, Ghent University Merelbeke Belgium

**Keywords:** burden of disease, cancer, cost‐of‐illness

## Abstract

**Background:**

Similar to many countries, Belgium experienced a rapid increase in cancer diagnoses in the last years. Considering that a large part of cancer types could be prevented, our study aimed to estimate the annual healthcare burden of cancer per site, and to compare cost with burden of disease estimates to have a better understanding of the impact of different cancer sites in Belgium.

**Methods:**

We used nationally available data sources to estimate the healthcare expenditure. We opted for a prevalence‐based approach which measures the disease attributable costs that occur concurrently for 10‐year prevalent cancer cases in 2018. Average attributable costs of cancer were computed via matching of cases (patients with cancer by site) and controls (patients without cancer). Years of life lost due to disability (YLD) were used to summarize the health impact of the selected cancers.

**Results:**

The highest attributable cost in 2018 among the selected cancers was on average €15,867 per patient for bronchus and lung cancer, followed by liver cancer, pancreatic cancer, and mesothelioma. For the total cost, lung cancer was the most costly cancer site with almost €700 million spent in 2018. Lung cancer was followed by breast and colorectal cancer that costed more than €300 million each in 2018.

**Conclusions:**

In our study, the direct attributable cost of the most prevalent cancer sites in Belgium was estimated to provide useful guidance for cost containment policies. Many of these cancers could be prevented by tackling risk factors such as smoking, obesity, and environmental stressors.

## 1 INTRODUCTION

Similar to many countries in the world, Belgium experienced a rapid increase in cancer diagnoses in the last years. In 2019, 80,524 persons were diagnosed with cancer and a total of 432,106 people had a cancer diagnosis in the previous 10 years.^1^ The increase in cancer incidence is largely attributed to population growth, the aging population, and increased exposure to risk factors (e.g. overweight and obesity, tobacco, exposure to human papillomavirus or hepatitis B or hepatitis C viruses) resulting in a high unmet need for effective and well‐tolerated treatments as well as early disease detection.^2^ Meeting these needs requires a substantial increase in public health and healthcare expenditure. For instance, an increasing number of cancer patients leads to an increase of healthcare expenditure for diagnostics and treatment, as new treatment modalities typically require additional healthcare spending.^3^ Nevertheless, the constrained healthcare budgets have increased the pressure to implement cost‐effective strategies,^2^ including the prevention of new cancer diagnoses. For this purpose, two main designs are possible for cost‐of‐illness studies: a prevalence‐based approach (in which new as well as pre‐existing illness in a given year is assessed) is more suitable for ascertaining the total current economic burden of a disease, whereas an incidence‐based approach (in which only new cases are included) is more useful for ascertaining the expected impact of a disease in the future (and its potential prevention). In addition, considering costs in the context of the disease burden—as captured by the disability adjusted life years (DALY) metric—allows comparisons of the economic and disease burden among cancer or chronic diseases in general and may facilitate health policy and decisions regarding resource allocation. Currently available studies on cost of cancer constitute estimates derived by European or multistate studies (OECD, WHO countries).^4^, ^5^ While these estimates can provide a broad (not tailored) overview of the health status in Belgium, it remains a question whether these estimates could be improved by making use of available national registry data. Targeted studies were conducted in Belgium dealing with specific site of the cancer,^6^, ^7^ not allowing for a comparison among cancer sites.

In order to better understand the economic burden of cancer in Belgium, our study aimed to estimate the annual incremental healthcare costs sustained by the healthcare system per cancer site (using a prevalence approach), and to compare cost with burden of disease estimates to have a better understanding of the impact of different cancer sites in Belgium.

## 2 METHODS

Our study concerns the linkage between a nationwide registry database and the national health insurance data: the Belgian Cancer Registry (BCR) collecting information on cancer diagnosis; and the Intermutualistic Agency (IMA) summarizing healthcare expenditure data. We opted for a prevalence‐based approach, which measures the disease attributable costs that occur concurrently for 10‐year prevalent cancer cases over a year of reference, 2018.^8^ Average attributable costs of cancer were computed via matching of cases (patients with cancer by site) and controls (patients without cancer). Years of life lost due to disability (YLD) were used to summarize the health impact of the selected cancers and were retrieved from the Belgian Burden of Disease study^1^ for the year 2018. YLD were also based on a 10‐year prevalence perspective.

### 2.1 Data sources

Data on cancer cases in Belgium were collected by the BCR, which is a population‐based registry regularly reporting on cancer patterns and trends in incidence and cancer survival. It is nationally representative and exhaustive, collecting data from the oncological care programs (clinical network) and pathology laboratories (pathological network).^9^ The recording of data (topography and morphology) is done using the International Classification of Diseases for Oncology third edition (ICD‐O‐3), which is combined into a ICD‐10 classification (International Classification of Diseases tenth edition). For the current study, we selected the ICD‐10 codes shown in Table 1 resulting in 21 cancer sites.^9^

**TABLE 1:** Selected cancer sites.

ICD‐10 code	Cancer location
C00‐C14; C30‐C32	Head and neck
C15‐C16.0	Esophagus
C16.1‐C16.09	Stomach
C18‐C19	Colon
C20	Rectum
C22	Liver
C23‐C24	Gallbladder and biliary tract
C25	Pancreas
C34	Bronchus and lung
C43	Malignant melanoma
C45	Mesothelioma
C50	Breast
C53	Cervix
C54	Corpus uteri
C56	Ovary
C61	Prostate
C62	Testis
C64	Kidney
C67	Bladder
C71‐C72	Central nervous system
C73	Thyroid

The cancer diagnosis by year and site, as well as whether there were additional cancer diagnoses in the 10 years before inclusion were retrieved from the BCR. These data were linked to the national health insurance data compiled by IMA for the year 2018 using the national registry number, which allows to identify each Belgian resident. Health insurance is compulsory in Belgium covering more than 98% of the population. The IMA database comprised reimbursed total healthcare costs, for every payment modality (directly paid by the health insurance, patients out‐of‐pocket and supplements). These expenditures included ambulatory care and reimbursed medicines purchased through pharmacies (over‐the‐counter pharmaceuticals excluded) and hospital care including medications administered at the hospital. The IMA database provided the healthcare cost for the year 2018 of the included cancer patients, as well as a control sample. For both samples, IMA provided data on individual age, sex, region of residence (i.e. Brussels‐Capital, Flemish, and Walloon Region) and reimbursement status. The latter refers to whether the person benefits from preferential reimbursement for healthcare use. In Belgium, this is referred to as BIM (bénéficiaire de l'intervention majorée) or OMNIO (increased reimbursement of medical costs for low income families) and it is granted to certain categories of people (mainly people with low incomes, beneficiaries of the social allowances, or elderly people with a low income). This was considered as a proxy for socioeconomic status. In addition to the variables above described, the IMA database includes information on the number of hospitalization visits for both controls and cases.

### 2.2 Study design

**FIGURE 1:**
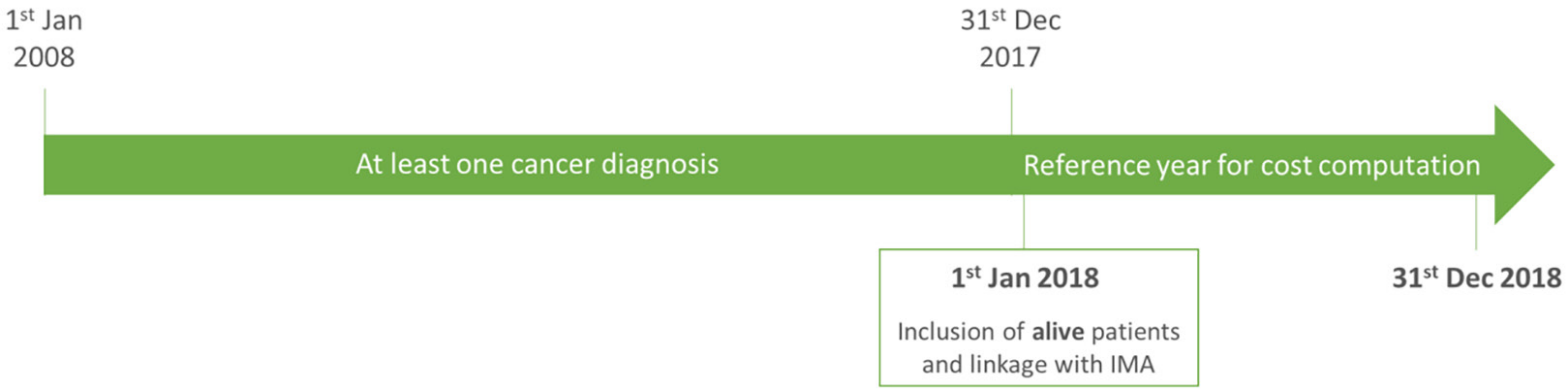
Selection of cancer cases.

Cancer patients were selected following a 10‐year prevalence perspective. Cancer cases were defined as being alive on January 1, 2018, and had at least one cancer diagnosis of the included cancer sites during the previous 10 years (Figure 1). After appropriate calculation of the sample size in order to achieve significant results, a maximum of 1435 cases was selected for each cancer site.

For each cancer case, four controls were assigned with the exact same age (in years), sex, region of residence, and reimbursement status. Controls were included if they were alive on January 1, 2018 and did not have a cancer diagnosis in the 10 years prior to January 1, 2018.

### 2.3 Statistical analysis

All analyses were performed in R version 1.4.1717.^10^ Descriptive data are provided as means and SDs or frequencies and percentages. Average incremental costs associated with cancer were estimated at the individual level for each cancer site using the method of standardization (also known as g‐computation).^11^, ^12^ This method estimates the marginal difference in costs for the subgroup being affected by one cancer site compared to the population that does not have any cancer diagnosis.

A multivariable regression was performed in the matched sample with average total healthcare costs as the dependent and the presence of a specific cancer site, age, sex, region of residence and reimbursement status as independent variables. A generalized linear regression was used with costs modeled with a negative binomial distribution. The estimated coefficients of the regression model were used to predict the healthcare costs for each respondent if the specific cancer was not present, keeping all other characteristics as observed. The difference between the two predicted costs was considered as the individual incremental cost of the specific cancer site. Finally, the attributable cost of the cancer site was computed as the average of the individual incremental costs for the specific cancer site. The confidence intervals (CI) of the incremental values were obtained using the qnorm function on the standard errors obtained from the regression models. Total incremental costs were computed by multiplying the mean incremental cost and the total number of cases in 2018 of each cancer site.

In addition to the incremental cost analysis, we analyzed the association of different variables that could affect the cost attributable to cancer (i.e. age, years since diagnosis, and number of cancer in different sites). For this purpose we run different univariate regression models with costs modeled with a negative binomial distribution.

We used charts to illustrate the association between YLD and average incremental cost per case for every cancer site.

## 3 RESULTS

In total, 138,226 people were included in the analysis—27,758 prevalent cancer cases and 110,468 controls. The two samples had similar sociodemographic characteristics as shown in Appendix Table A1. An initial descriptive analysis of healthcare utilization showed that people affected by cancer had significantly higher healthcare costs than people without cancer (Table 2). Ambulatory costs, including reimbursable medications purchased in pharmacies, were the highest contributor to the total healthcare costs (Table 2). Cases also had a significantly higher number of hospitalizations than controls in the year 2018. Some additional information on the sample of cases included in the study showed that on average cases were diagnosed 4.46 years before inclusion and that the great majority had only one cancer (88.5%), whereas 10.5% was diagnosed with two cancers and 1% with three or more cancer sites.

**TABLE 2:** Descriptive analysis of healthcare costs, hospitalizations, years since diagnosis, and total number of cancers for controls and cases for 2018.

	Controls	Cases	*p*‐value^a^
*N*	110,468	27,758	
Hospitalization costs, mean (SD)	1850.7 (7396.5)	5079.9 (12,943.7)	<0.001
Ambulatory costs, mean (SD)	2994.4 (5832.9)	6986.5 (12,431.5)	<0.001
Other costs, mean (SD)	162.9 (1063.0)	341.3 (1392.4)	<0.001
Total healthcare cost, mean (SD)	5007.9 (10,802.2)	12,407.7 (20,025.9)	<0.001
Total number of hospitalizations, mean (SD)	0.48 (1.3)	2.29 (4.7)	<0.001
Out of which day‐hospital	0.22 (0.98)	1.62 (4.3)	<0.001
Years since diagnosis, median (IQR)	NA	4 (2–7)	NA
Total number of cancers, *N* (%)			
1	NA	24,566 (88.5)	NA
2	NA	2907 (10.5)	
3 or more	NA	285 (1.0)	

^a^ Chi‐squared test for categorical variables and one‐way ANOVA test for continuous variables.

### 3.1 Average incremental cost of cancer

Table 3 shows the results of the average incremental cost by cancer site. The highest attributable cost among the selected cancers was on average €15,867 per patient for bronchus and lung cancer in 2018, followed by liver cancer, pancreatic cancer, and mesothelioma. In 2018, the cancer types for which the average incremental cost per patient was the lowest were uterine, prostate, and thyroid cancer.

**TABLE 3:** Average incremental costs and 95% confidence intervals by cancer site.

Cancer site	Average incremental cost	95% Confidence interval
Bronchus and lung	15,904	15,686–16,122
Liver	15,313	15,123–15,503
Pancreas	14,046	13,906–14,186
Mesothelioma	13,041	12,623–13,459
Esophagus	12,158	12,020–12,296
Ovary	11,027	10,911–11,143
Central nervous system	9943	9796–10,090
Gallbladder and biliary tract	9883	9553–10,213
Head and neck	8299	8159–8439
Bladder	7294	7160–7428
Kidney	6633	6568–6698
Rectum	6309	6171–6447
Stomach	6095	5943–6247
Cervix uteri	4216	4111–4321
Colon	4030	3925–4135
Malignant melanoma of skin	3781	3660–3902
Breast	3589	3491–3687
Corpus uteri	2880	2831–2929
Prostate	2414	2372–2456
Thyroid gland	2264	2214–2314
Testis	1278	1226–1330

The analysis of the association between average incremental cost attributable to cancer and different covariates (Table 4) showed that the cancer patients between 65 and 74 years had the highest cost of cancer (70.3% higher than the youngest age group [<35 years]). People affected by cancer since less than 3 years had the highest incremental cost compared to people with a cancer diagnosis since more than 8 years: 42% higher for 1 year or less and 22.9% for 2–3 years of diagnosis. Having cancer in multiple sites was also associated with higher costs (14.8% higher for two sites and 16.7% higher for 3 or more sites).

**TABLE 4:** Cost ratios and 95% confidence intervals for univariate analysis of average incremental cost attributable to cancer.

	*N* (%)	Cost ratios	95% confidence intervals
Age (years)			
<35	1040 (3.7)	Ref.	
35–44	1761 (6.3)	0.986	0.933–1.044
45–54	2994 (10.8)	1.314	1.25–1.382
55–64	5549 (20.0)	1.599	1.526–1.676
65–74	7923 (28.5)	1.703	1.627–1.784
75–84	5957 (21.5)	1.644	1.569–1.723
85+	2534 (9.1)	1.459	1.388–1.535
Years since diagnosis			
1 year or less	5361 (19.3)	1.420	1.389–1.452
From 2 to 3 years	7281 (26.2)	1.229	1.203–1.256
From 4 to 5 years	5305 (19.1)	1.099	1.073–1.125
From 6 to 7 years	4461 (16.1)	1.057	1.031–1.084
More than 8 years	5350 (19.3)	Ref.	
Total number of cancers			
1	24,566 (88.5)	Ref.	
2	2907 (10.5)	1.148	1.123–1.173
3+	285 (1.0)	1.167	1.092–1.246

Cost ratios are the exponential of the coefficients resulting from the univariate analysis. They should be interpreted as increasing by one level the independent covariates times the mean attributable costs by the cost ratio.

### 3.2 Years lived with disability and incremental healthcare cost

**FIGURE 2:**
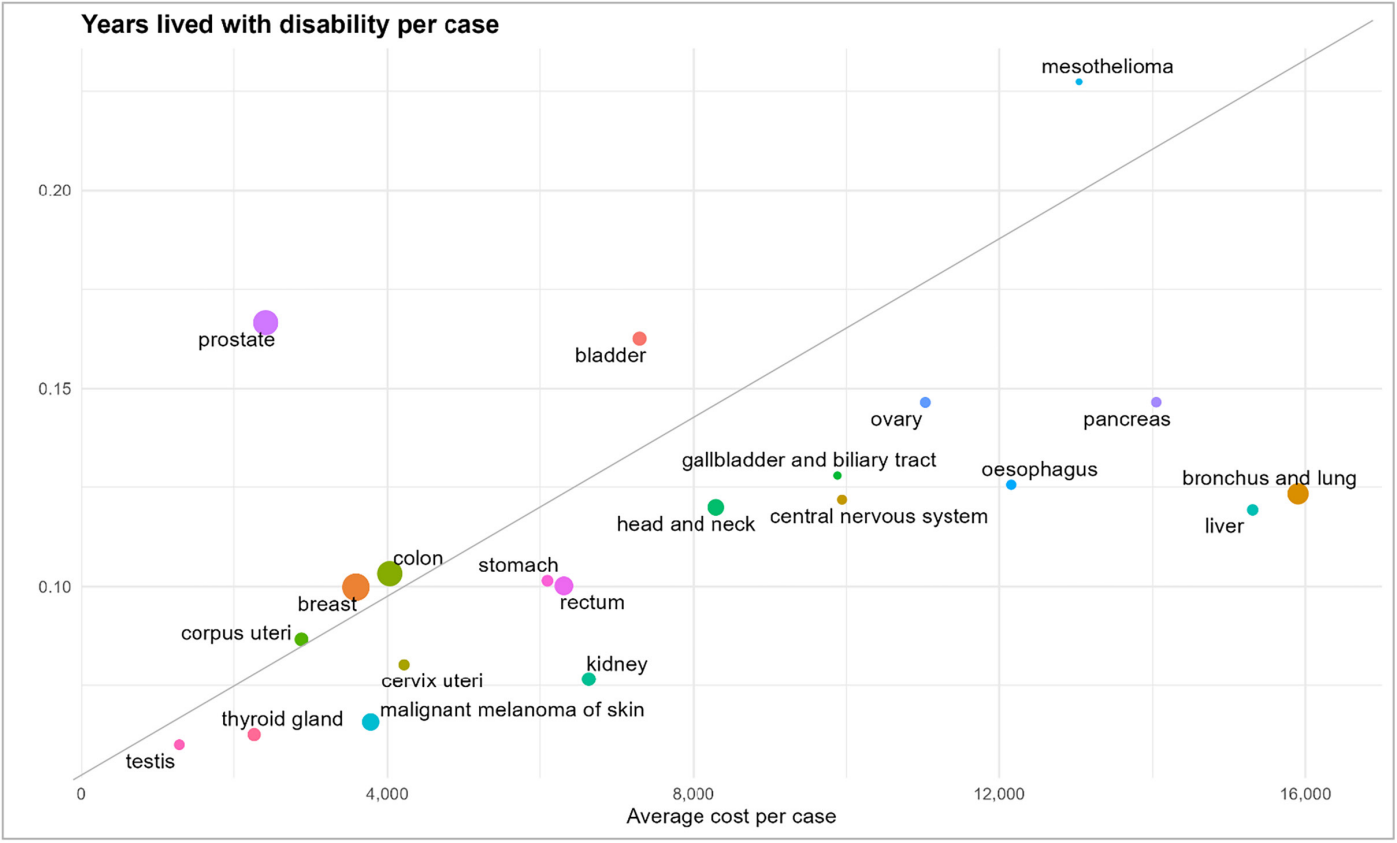
Years lived with disability per case and average incremental healthcare cost per case by cancer site.

Figure 2 shows that for most cancer sites the YLD estimates per case are commensurate with the incremental healthcare cost per case (they lie around the 45° line). In particular, the cancer sites that are in the left‐lower quadrant have a low impact on the YLD of individuals and a lower cost per patient (e.g. breast, stomach, corpus uteri, thyroid cancer). On the other hand, mesothelioma shows a high YLD and high cost per case. Few cancers showed a disproportion of YLD and cost per case.

For instance, people living with prostate cancer have a high burden but the healthcare cost spent for these patients is not proportionate to their burden. Cancer sites lying on the right‐lower quadrant have a higher cost per case than their YLD burden.

**FIGURE 3:**
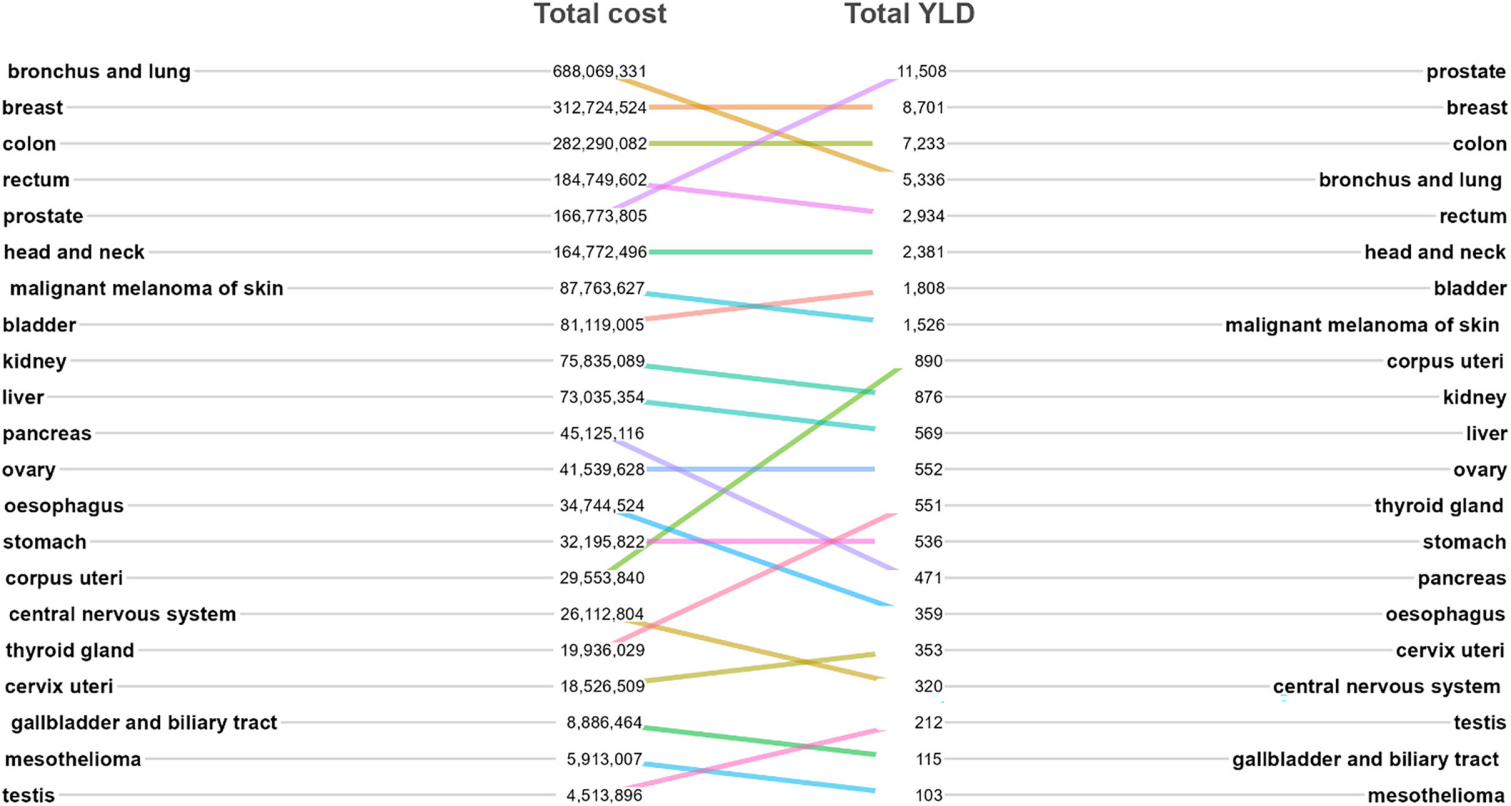
Comparison of total years of life lived with disability and total costs.

Data on the total healthcare cost and YLD burden are summarized in Figure 8. When looking at the total cost, bronchus and lung cancer was by far the most costly cancer site with almost €700 million spent in 2018. Lung cancer was followed by breast and colorectal cancer that costed more than €300 million each in 2018 (Figure 3). In the case of mesothelioma, the comparison of total YLD and costs showed a different picture: mesothelioma has the lowest total health and economic burden.

## 4 DISCUSSION

We estimated the direct attributable cost of 21 different cancer sites in Belgium, using data collected at the national level. Ambulatory visits, including medication costs, appeared to be the highest contributor to the total healthcare expenditure of people with a cancer diagnosis. In 2018, people with a cancer diagnosis had a total direct cost 2.5 times higher than people without cancer. Bronchus and lung cancer, liver cancer, pancreas cancer, and mesothelioma were among the sites with the highest direct cost. This might be explained by the relatively low survival and duration of the disease, leading to have in our sample a combination of many “new” cases in 2018, with a higher cost for starting a treatment, and cases in a final stage of cancer in 2018, with higher end‐of‐life costs. We also investigated the association between average incremental cost attributable to cancer and different covariates that could impact cost of cancer. Costs decreased with the years since diagnosis and increased if cancer was present in more than one site.

Comparing the average cost per case highlights the cancer sites (bronchus and lung cancer, liver cancer, pancreatic cancer, and mesothelioma) that are more costly regardless of the number of people that are affected by them. We were also interested in investigating which cancer sites have the highest burden on the total healthcare cost reimbursed by the Belgian public health insurance funds. Bronchus and lung cancer was by far the most costly cancer site, with a cost two times higher than breast and colorectal cancer (respectively the second and third most costly cancer sites at population level). To date, only studies referring to the total cost of cancer (without distinction among cancer site)^3^ or studies focusing on the cost of one specific cancer site^13^ can be found in the literature. In the Flemish region, the direct cost attributable to breast cancer was estimated to amount to €12,037 per patient over a 6‐year period.^7^ A study comparing the cost of different cancer sites in the United States showed that the total national expenditures for care of breast cancer was the highest, followed by colorectal, lung, and prostate cancer,^14^ revealing a similar rank to the one of our study.

Our study adopted a prevalence approach to assess the total economic burden of a cancer site in a specific year. This provides decision makers with a picture of the global burden and the areas where cost containment policies would have the greatest impact,^15^ which is informative to design cost containment policies. This approach should not be confused with the incidence approach where lifetime costs are computed for the cases that occur during the defined base incident year, and that are more suitable for measuring the potential savings from preventive interventions.^8^ Within our approach, caution is nonetheless warranted when contrasting costs between prevalent cases versus controls.^16^, ^17^ First, under our definition, prevalent cases have survived until January 1, 2018, in spite of their cancer. This may lead to selection bias since the group of cancer cases likely contains fewer people who have poor health for reasons other than their cancer (e.g., lifestyle) than controls. Indeed, such cases are at greater risk of death by January 1, 2018 than similar people in the control group, due to their cancer. Since people with poor health likely also have higher medical costs, such selection bias may have led to underestimation of the attributable costs. This is contrary to the confounding bias expected as a result of not having matched cases and controls on key lifestyle factors. The data available do not allow to neither include these factors in the analysis nor quantify the bias derived from them. Second, the comparison of attributable costs between cancer sites is partly influenced by the fact that cases may be in different stages of disease between different cancer sites, as a result of cancer site‐specific mortality rates.

Policy actions addressing the burden of cancer and its impacts should be twofold; first, addressing the onset and recovery of cancer in Belgium, and secondly reducing its association with adverse health outcomes (e.g., complications) that result in a higher healthcare use. These policies are needed to target cancer both at the individual level and at the population level, as highlighted by our research. For instance, we showed that the cost of bronchus and lung cancer is the highest and it is one of the most prevalent in Belgium. Mesothelioma was the cancer with the highest YLD per case rate and among the cancers with the highest cost per case, but the lowest total health and economic burden due to its low prevalence. This highlights the need to look at the problem both from an individual and population perspective. It was also revealed that some cancer sites have a low health burden and healthcare cost per case. In the case of breast and cervical cancer, these might be associated with the success of screening measures achieving an early detection and consequentially a lower burden. Cost of current care (which is considered here) represents one element to be considered in cost containment policies. A higher cost can also be associated with a higher cost for innovative treatments resulting in increased survival (and in the long run in a lower disability burden and increased productivity). Ideally it should be taken into account together with other factors: potential and cost‐effectiveness of screening, new therapies that become available. In addition, higher costs and higher YLD might correspond to a longer survival of people living with cancer. Higher costs could be caused by new treatments that improved survival and increasing time of disease monitoring.

We would also like to draw the attention to the fact that many of the high‐ranking cancers are very strongly linked to risk factors such as smoking, alcohol, lack of physical activity, obesity, and environmental stressors. This reinforces the message that the best investment for health policy makers to reduce cancer and cancer costs is to take actions to reduce these risk factors at the population level.

### 4.1 Strengths and limitations

Our analysis included national claims data collected at the population level and include inpatient, outpatient healthcare, and prescribed medication. Considering that indirect costs are difficult to track and quantify, our analysis focused on the direct cost of cancer. Indirect costs, including loss of productivity and informal caregiving costs, represent an important part of cost for patients with cancer that clearly add up to the total cost of cancer. Some limitations are attributed to the possible failure to control sufficiently for confounding and selection bias. This is due to the limited amount of available data in our data sources. For example, neither BCR nor IMA has indication on lifestyle risk factors and complete information on comorbidities.

Our results should be interpreted as a snapshot of healthcare costs related to cancer in a specific year based on a 10‐year prevalence approach rather than lifetime costs of cancer (incidence‐based approach).

## 5 CONCLUSION

In our study, the direct attributable cost of the most prevalent cancer sites in Belgium was estimated to provide useful guidance for cost containment policies. The highest healthcare costs occurred in bronchus and lung cancer patients, for which both the total and the average cost per patient were the highest. Mesothelioma was the cancer with the highest YLD per case rate and among the cancers with the highest cost per case. On the other hand, breast and cervical cancer showed a low health and economic burden per case. These might be associated with the success of screening measures achieving an early detection and consequentially a lower burden.

## AUTHOR CONTRIBUTIONS

**Vanessa Gorasso:** Conceptualization (equal); data curation (lead); formal analysis (lead); investigation (lead); methodology (equal); project administration (lead); software (lead); writing – original draft (lead). **Stefanie Vandevijvere:** Supervision (equal); validation (equal); writing – review and editing (equal). **Johan Van der Heyden:** Validation (equal); writing – review and editing (equal). **Ingrid Pelgrims:** Validation (equal); writing – review and editing (equal). **Henk Hilderink:** Supervision (equal); validation (equal); writing – review and editing (equal). **Wilma Nusselder:** Validation (equal); writing – review and editing (equal). **Claire Demoury:** Validation (equal); writing – review and editing (equal). **Masja Schmidt:** Validation (equal); writing – review and editing (equal). **Stijn Vansteelandt:** Formal analysis (equal); methodology (equal); validation (equal); writing – review and editing (equal). **Delphine De Smedt:** Conceptualization (equal); supervision (equal); validation (equal); visualization (equal); writing – review and editing (equal). **Brecht Devleesschauwer:** Conceptualization (equal); formal analysis (equal); methodology (equal); supervision (equal); validation (equal); writing – review and editing (equal).

## FUNDING INFORMATION

VG received funds from Sciensano for this work.

## CONFLICT OF INTEREST STATEMENT

All authors declare that they have no conflict of interest.

## ETHICS STATEMENT

The current study was approved by the data protection committee in Belgium (approval no. CSI/CSSS/21/270).

## Supporting information


Table S1.

## Data Availability

The data that support the findings of this study are available from IMA but restrictions apply to the availability of these data, which were used after approval for the current study, and so are not publicly available.

## APPENDIX A

**TABLE A1:** Total costs, prevalence, total and per case years of life lived with disability.

Cancer site	Total cost in Euros	Prevalence^a^	Years of life lived with disability per case	Total years of life lived with disability^a^
Bladder	81,119,005	11,121	0.163	1808
Breast	312,724,524	87,134	0.100	8701
Bronchus and lung	688,069,331	43,264	0.123	5336
Central nervous system	26,112,804	2626	0.122	320
Cervix uteri	18,526,509	4394	0.080	353
Colon	282,290,082	70,047	0.103	7233
Corpus uteri	29,553,840	10,262	0.087	890
Gallbladder and biliary tract	8,886,464	899	0.128	115
Head and neck	164,772,496	19,854	0.120	2381
Kidney	75,835,089	11,433	0.077	876
Liver	73,035,354	4770	0.119	569
Malignant melanoma of skin	87,763,627	23,212	0.066	1526
Mesothelioma	5,913,007	453	0.227	103
Esophagus	34,744,524	2858	0.126	359
Ovary	41,539,628	3767	0.147	552
Pancreas	45,125,116	3213	0.147	471
Prostate	166,773,805	69,086	0.167	11,508
Rectum	184,749,602	29,284	0.100	2934
Stomach	32,195,822	5282	0.101	536
Testis	4,513,896	3532	0.060	212
Thyroid gland	19,936,029	8806	0.063	551

^a^ Derived from Gorasso et al.^1^

## References

[1] Gorasso V , Silversmit G , Arbyn M , et al. The non‐fatal burden of cancer in Belgium, 2004‐2019: a nationwide registry‐based study. BMC Cancer. 2022;22(1):58. doi:10.1186/s12885-021-09109-4 35026995 PMC8756629

[2] Schlueter M , Chan K , Lasry R , Price M . The cost of cancer—a comparative analysis of the direct medical costs of cancer and other major chronic diseases in Europe. PloS One. 2020;15(11):e0241354. doi:10.1371/journal.pone.0241354 33175865 PMC7657541

[3] Hofmarcher T , Lindgren P , Wilking N , Jönsson B . The cost of cancer in Europe 2018. Eur J Cancer. 2020;129:41‐49. doi:10.1016/j.ejca.2020.01.011 32120274

[4] Tarricone R . Cost‐of‐illness analysis. What room in health economics? Health Policy. 2006;77(1):51‐63. doi:10.1016/j.healthpol.2005.07.016 16139925

[5] Wilking NE , Hofmarcher T , Lindgren P , Jönsson B . The burden and direct cost of cancer in Europe (EU‐28). J Clin Oncol. 2016;34(15):6618. doi:10.1200/JCO.2016.34.15_suppl.6618

[6] Pil L , Hoorens I , Vossaert K , Brochez L , Annemans L . The Impact of Skin Cancer in Belgium and the Cost‐Effectiveness of Prevention. KCE; 2016. 10.1016/j.ypmed.2016.10.005 27713103

[7] Broekx S , Hond ED , Torfs R , et al. The costs of breast cancer prior to and following diagnosis. Eur J Health Econ. 2011;12(4):311‐317. doi:10.1007/s10198-010-0237-3 20306109

[8] Larg A , Moss JR . Cost‐of‐illness studies: a guide to critical evaluation. Pharmacoeconomics. 2011;29(8):653‐671. doi:10.2165/11588380-000000000-00000 21604822

[9] Belgian Cancer Registry . Cancer Burden in Belgium 2004–2017. 2020 [Online]. https://kankerregister.org/media/docs/CancerBurdenfeb2020reduced.pdf

[10] R Core Team . R: A Language and Environment for Statistical Computing. R Foundation for Statistical Computing; 2021. https://www.R‐project.org/

[11] Keiding N , Clayton D . Standardization and control for confounding in observational studies: a historical perspective. Stat Sci. 2014;29(4). doi:10.1214/13-STS453

[12] Snowden JM , Rose S , Mortimer KM . Implementation of G‐computation on a simulated data set: demonstration of a causal inference technique. Am J Epidemiol. 2011;173(7):731‐738. doi:10.1093/aje/kwq472 21415029 PMC3105284

[13] Henderson RH , French D , Maughan T , et al. The economic burden of colorectal cancer across Europe: a population‐based cost‐of‐illness study. Lancet Gastroenterol Hepatol. 2021;6(9):709‐722. doi:10.1016/S2468-1253(21)00147-3 34329626

[14] Mariotto AB , Enewold L , Zhao J , Zeruto CA , Yabroff KR . Medical care costs associated with cancer survivorship in the United States. Cancer Epidemiol Biomark Prev. 2020;29(7):1304‐1312. doi:10.1158/1055-9965.EPI-19-1534 PMC9514601 32522832

[15] Jo C . Cost‐of‐illness studies: concepts, scopes, and methods. Clin Mol Hepatol. 2014;20(4):327‐337. doi:10.3350/cmh.2014.20.4.327 25548737 PMC4278062

[16] Hernán MA . Counterpoint: epidemiology to guide decision‐making: moving away from practice‐free research. Am J Epidemiol. 2015;182(10):834‐839. doi:10.1093/aje/kwv215 26507306 PMC4634308

[17] Danaei G , Tavakkoli M , Hernán MA . Bias in observational studies of prevalent users: lessons for comparative effectiveness research from a meta‐analysis of statins. Am J Epidemiol. 2012;175(4):250‐262. doi:10.1093/aje/kwr301 22223710 PMC3271813

