# Supplementary material for: The incremental healthcare cost associated with cancer in Belgium: A registry‐based data analysis
Source: Cancer Med. 2024 Jan 24;13(3):e6659. doi: 10.1002/cam4.6659 (PMC10905540; doi:10.1002/cam4.6659)
Supplement: Supplementary file 1 — Table S1. [file CAM4-13-e6659-s001.pdf]

The incremental healthcare cost associated with cancer in Belgium: a registry-based data analysis.

Vanessa Gorasso, Stefanie Vandevijvere, Johan Van der Heyden, Ingrid Pelgrims, Henk Hilderink, Wilma Nusselder, Claire Demoury, Masja Schmidt, Stijn Vansteelandt, Delphine De Smedt, Brecht Devleesschauwer

***Corresponding author:** Vanessa Gorasso – Health Information, Department of epidemiology and public health, Sciensano, Rue J Wytsman 14, 1050 Brussels, Belgium.

Supplementary information

Appendix table 1: Sample characteristics after matching

		Controls	Cases	p-value
**N**		110,468	27,758	
**Age – mean (SD)**		65.88 (15.0)	65.91 (15.0)	0.742
**Sex – N (%)**	Male	56,336 (51.0)	14,176 (51.1)	0.834
	Female	54,132 (49.0)	13,582 (48.9)	
**Region - N (%)**	Brussels	8032 (7.3)	2020 (7.3)	1
	Flanders	65,660 (59.4)	16,499 (59.4)	
	Wallonia	36,476 (33.0)	9164 (33.0)	
	Missing	300 (0.3)	75 (0.3)	
**Reimbursement status– N (%)**	No particular status	81,276 (73.6)	20,416 (73.5)	0.941
	Increased reimbursement*	29,192 (26.4)	7342 (26.5)	

*In Belgium this is referred to as BIM or OMNIO status. It grants a higher reimbursement of healthcare costs to certain categories of people (mainly people with low incomes, beneficiaries of social allowances or elderly people with a low income)
